# Engineered biosynthesis of bacteriochlorophyll *b* in *Rhodobacter sphaeroides*

**DOI:** 10.1016/j.bbabio.2014.07.011

**Published:** 2014-10

**Authors:** Daniel P. Canniffe, C. Neil Hunter

**Affiliations:** Department of Molecular Biology and Biotechnology, University of Sheffield, Firth Court, Western Bank, Sheffield S10 2TN, UK

**Keywords:** BChl, bacteriochlorophyll, RC, reaction center, LH, light-harvesting complex, Chl, chlorophyll, E, ethyl, Chlide, chlorophyllide, V, vinyl, 8VR, C8-vinyl reductase, COR, chlorophyllide oxidoreductase, BChlide, bacteriochlorophyllide, Bacteriochlorophyll, Chlorophyll, Chlorophyllide oxidoreductase, Photosynthesis, Pathway engineering

## Abstract

Bacteriochlorophyll *b* has the most red-shifted absorbance maximum of all naturally occurring photopigments. It has a characteristic ethylidene group at the C8 position in place of the more common ethyl group, the product of a C8-vinyl reductase, which is carried by the majority of chlorophylls and bacteriochlorophylls used in photosynthesis. The subsequent and first step exclusive to bacteriochlorophyll biosynthesis, the reduction of the C7 = C8 bond, is catalyzed by chlorophyllide oxidoreductase. It has been demonstrated that the enzyme from bacteriochlorophyll *a*-utilizing bacteria can catalyze the formation of compounds carrying an ethyl group at C8 from both ethyl- and vinyl-carrying substrates, indicating a surprising additional C8-vinyl reductase function, while the enzyme from organisms producing BChl *b* could only catalyze C7 = C8 reduction with a vinyl substrate, but this product carried an ethylidene group at the C8 position. We have replaced the native chlorophyllide oxidoreductase-encoding genes of *Rhodobacter sphaeroides* with those from *Blastochloris viridis*, but the switch from bacteriochlorophyll *a* to *b* biosynthesis is only detected when the native conventional C8-vinyl reductase is absent. We propose a non-enzymatic mechanism for ethylidene group formation based on the absence of cellular C8-vinyl reductase activity.

## Highlights

• We engineer the production of a foreign photopigment in *Rhodobacter sphaeroides*.
• Native COR-encoding genes are replaced with those from *Blastochloris viridis*.
• Bacteriochlorophyll *b* is produced upon additional deletion of conventional 8VR.
• We propose that loss of 8VR activity by COR leads to ethylidene bond formation.

## 1 Introduction

**Fig. 1:**
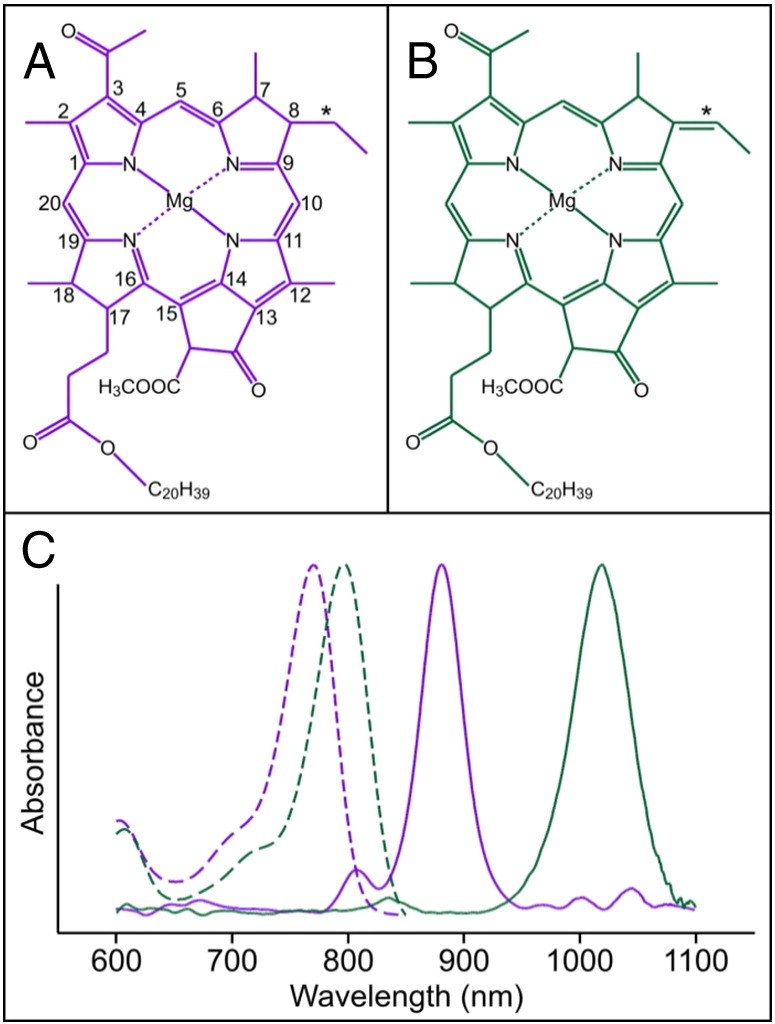
Structures and absorption properties of BChls *a* and *b.* (A) IUPAC numbered chemical structure of BChl *a*. The ethyl group at C8 is indicated by an asterisk. (B) Chemical structure of BChl *b*. The ethylidene group at C8 is indicated by an asterisk. (C) Absorption spectra of unbound BChls *a* (dashed purple) and *b* (dashed green), and RC-LH1 complexes containing BChls *a* or *b* from *Rba. sphaeroides* (solid purple) and *Blc. viridis* (solid green), respectively.

Bacteriochlorophyll (BChl) *b*, the most strongly red-shifted (bacterio)chlorin used for light harvesting, was first discovered in 1963 as the major photosynthetic pigment of a *Rhodopseudomonas* sp. photosynthetic bacterium [1]. BChl *b* differs from BChl *a*, the most abundant bacteriochlorin, by the presence of an ethylidene group rather than an ethyl (E) group at the C8 position [2] (Fig. 1A and B), which shifts the Q_y_ absorption band of the unbound pigment 25 nm to the red (from 770 to 795 nm in methanol) [3] (Fig. 1C). BChl *b* is found in the photosynthetic reaction center (RC)/light-harvesting (LH) 1 complex of *Blastochloris* (*Blc*.) *viridis* (formerly *Rhodopseudomonas viridis*), first isolated and studied by Drews and Giesbrecht [4], which is able to absorb solar energy approximately 150 nm further into the infrared than an analogous BChl *a*-containing complex (Fig. 1C). The RC from *Blc. viridis* was the first membrane protein complex to have its 3D structure determined by X-ray crystallography [5]. The spectral characteristics of BChls allow photosynthetic bacteria to absorb solar energy that is not absorbed by the chlorophyll (Chl)-utilizing cyanobacteria and algae found higher in the water column [6]. The very large red shifts seen for BChl *b*-containing LH1 complexes, which extend absorption to 1023 nm, sample a region of the solar spectrum well to the red of BChl *a*-containing LH1 complexes, which absorb in the 800- to 900-nm region. A recent review proposed a re-engineered photosynthetic apparatus for energy capture in which one of the two photosystems found in plants and cyanobacteria was modified so that it could utilize far-red light, while the other photosystem retained the ability to absorb light in the 650- to 700-nm region [7]. Accomplishing such modifications requires control over pigment biosynthesis and photosystem assembly; in this work, we explore the potential of re-routing the biosynthesis of BChl *a* towards the formation of BChl *b.*

**Fig. 2:**
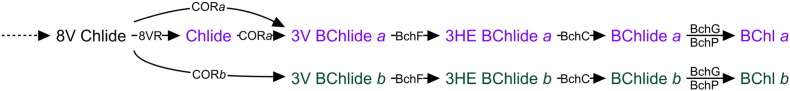
Biosynthetic pathways of BChls *a* and *b* from the precursor 8V Chlide. Pathways highlighted in purple and green are those that utilized by *Rba. sphaeroides* and *Blc. viridis*, respectively.

BChls *a* and *b* share a common biosynthetic pathway with Chls up to the precursor chlorophyllide (Chlide) (Fig. 2). The E group at the C8 position of Chls and BChl *a* results from the reduction of a vinyl (V) group on Chlide, catalyzed by an 8V reductase (8VR) [8], [9], [10], [11], while the first step in true bacteriochlorin biosynthesis, the reduction of the C7 = C8 of Chlide, is catalyzed by Chlide oxidoreductase (COR) [12], [13], [14], [15]. Recently, both *in vitro* assays using purified COR components (BchX, BchY and BchZ proteins) [16] and genetic complementations [17] have been used to demonstrate that that the enzymes from BChl *a*-utilizing bacteria (COR*a*) can catalyze the formation of 8E bacteriochlorophyllide (BChlide) from both 8E and 8V Chlide substrates, indicating a surprising additional 8VR function of the enzyme. However, an *in vitro* assay using the COR from *Blc. viridis* (COR*b*) indicated that this form of the enzyme was only able to catalyze BChlide formation with an 8V Chlide substrate, but this product, found to carry an ethylidene group at the C8 position, was BChlide *b* rather than BChlide *a* [16]. No product was formed when COR*b* was incubated with 8E Chlide, suggesting strict substrate specificity, correlating with the lack of an 8VR-encoding gene in the sequenced genomes of BChl *b*-producing organisms [16].

The purple phototrophic bacterium *Rhodobacter* (*Rba*.) *sphaeroides*, which utilizes BChl *a* in both its light-absorbing antennae and reaction centers, is a model organism for the study of photosynthesis and pigment biosynthesis due to its ability to grow chemotrophically in the dark, allowing deletion of genes involved in these processes [18]. The genome of *Rba. sphaeroides* contains genes encoding both a conventional 8VR (*bciA*) and COR*a*, a mechanism possibly used to ensure that only BChl carrying an 8E group is synthesized [11], [17], [19].

We have replaced the native COR*a*-encoding genes of *Rba. sphaeroides* with those encoding the *Blc. viridis* COR*b*. Interestingly, our results demonstrate that the COR*b* replacement mutant retained the ability to synthesize BChl *a*, while *b*-type bacteriochlorins could not be detected. This strain was able to photosynthesize, albeit at a reduced rate when compared to the wild type. However, the genetic replacement performed in a strain lacking the conventional 8VR resulted in the biosynthesis of BChl *b*, yet this second mutation culminated in the loss of photosynthetic viability. Our results lead us to postulate that, rather than catalyzing ethylidene group formation, COR*b* has lost 8VR activity. We predict that after the enzymatic reduction of C7 = C8, the resulting 8V bacteriochlorin (the existence of which has never been detected in nature) will spontaneously isomerize to the more stable *b*-type.

## 2 Materials and methods

### 2.1 Growth of described strains

*Rba. sphaeroides* strains were grown microoxically in the dark in a rotary shaker or phototrophically under illumination (90 μmol of photons · m^− 2^·s^− 1^) at 34 °C in liquid M22 + medium [20] supplemented with 0.1% casamino acids.

*Blc. viridis* was grown phototrophically in N_2_-sparged sodium succinate medium 27 (N medium) [21] under illumination (100 μmol of photons·m^− 2^·s^− 1^) at 30 °C as described by Lang and Oesterhelt [22].

*Escherichia coli* strains JM109 [23] and S17-1 [24] transformed with pK18*mobsacB* plasmids were grown in a rotary shaker at 37 °C in LB (Luria–Bertani) medium supplemented with 30 μg·ml^− 1^ kanamycin. All strains and plasmids used in the present study are listed in Supplementary Table 1.

### 2.2 Construction of mutants of *Rba. sphaeroides*

The *Rba. sphaeroides bchX*, *bchY* and *bchZ* genes were deleted using the allelic exchange vector pK18*mobsacB* [25]. Sequences up- and downstream of the three clustered genes were amplified with UpF (5′-CCGGAATTCCCGAAACTGATCGACAGTCTGG-3′; *Eco*RI restriction site underlined) and UpR (5′-CATGATCGCTCATTGCGTCATGCGGTGGCCCTCCAAT CC-3′), and DownF (5′-GGCCACCGCATGACGCAATGAGCGATCATGCCGTCAACACG-3′) and DownR (5′-CCCAAGCTTCGTGGGTTACGGTAAGGCACC-3′; *Hin*dIII restriction site underlined) primers, respectively. The up- and downstream PCR products were fused by overlap extension PCR, and the resulting product was digested with *Eco*RI and *Hin*dIII restriction enzymes and ligated into cut pK18*mobsacB*. Sequenced clones were conjugated into *Rba. sphaeroides* from *E. coli* S17-1, and transconjugants in which the clone had integrated into the genome by homologous recombination were selected on M22 + medium supplemented with kanamycin. Transconjugants that had undergone a second recombination event were then selected on M22 + supplemented with 10% (w/v) sucrose, lacking kanamycin. Sucrose-resistant kanamycin-sensitive colonies had excised the allelic exchange vector through the second recombination event [26]. The deletion of *bchX*, *bchY* and *bchZ* was confirmed by colony PCR using CheckF (5′-CCGCTCGATCTACGATGCGTC-3′) and CheckR (5′-CAGCCATGCTATCCTCCGG-3′) primers.

The *bchX*, *bchY* and *bchZ* genes from *Blc. viridis* were integrated into the genomes of *Rba. sphaeroides* strains at their original loci in strains that lack their native COR-encoding genes. A fragment of DNA containing the 3′ end of the *Rba. sphaeroides bchC* gene, the COR-encoding genes from *Blc. viridis* and the *Rba. sphaeroides pufQ* gene was synthesized by Genewiz (South Plainfield, NJ, USA). The sequence was edited to mimic the intergenic regions found between the *Rba. sphaeroides* COR-encoding genes, and silent base changes were introduced to remove restriction sites required for subcloning and to retain the essential upstream promoter region [27], ribosome binding site and start codon for *pufQ* (Supplementary Fig. 1). The synthesized fragment was sub-cloned into pK18*mobsacB* and conjugated into Δ*bchXYZ* background strains of *Rba. sphaeroides* as detailed above. Integration of the synthetic sequence, mediated by the presence of the *bchC* and *pufQ* sequences, was confirmed by colony PCR using the CheckF and CheckR primers detailed above.

### 2.3 Extraction of pigments

Pigments were extracted from cell pellets after washing in 20 mM HEPES pH 7.2 or from clarified growth medium by adding 9 pellet volumes of 0.2% (v/v) ammonia in methanol, vortex-mixing for 30 s and incubating on ice for 20 min. The extracts were clarified by centrifugation (15000*g* for 5 min at 4 °C), and the supernatants were immediately analyzed on an Agilent 1200 HPLC system.

### 2.4 Analysis of pigments by HPLC

BChl extracts were separated on a Supelco Discovery HS C18 reverse-phase column (5 μm particle size, 120 Å pore size, 250 x 4.6 mm) using a method modified from that of Frigaard *et al.* [28]. Solvents A and B were 42:33:25 (v/v/v) methanol/acetonitrile/H_2_O and 39:31:30 (v/v/v) methanol/acetonitrile/ethyl acetate, respectively. Pigments were eluted at 1 ml·min^− 1^ at 40 °C on a linear gradient of 70%–85% solvent B over 20 min, increasing to 100% to wash the column. Elution of BChl *a* and BChl *b* species were monitored by checking absorbance at 770 nm and 795 nm, respectively.

Chlide species were separated on a YMC30 C30 reverse-phase column (3 μm particle size, 250 × 4.6 mm) using a method modified from that of Kruk and Myśliwa-Kurdziel [29] as described previously [19].

## 3 Results and discussion

**Fig. 3:**
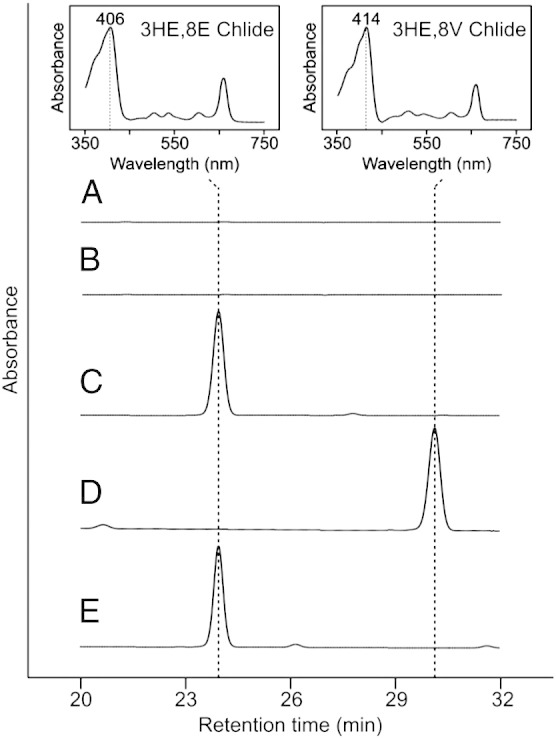
HPLC analysis of precursor pigments extracted from *Rba. sphaeroides* strains. HPLC elution profiles of extracts from (A) WT, (B) Δ*bciA*, (C) Δ*bchXYZ*, (D) Δ*bciA*/Δ*bchXYZ* and (E) mutant N22. Retention times of 23.9 and 30.2 min, and Soret absorption maxima at 406 and 414 nm (inset), are indicative of 3HE,8E Chlide and 3HE,8V Chlide, respectively, in the HPLC solvents. Traces are normalized to major peak height for clarity.

Due to the high sequence similarity between the COR-encoding genes from *Rba. sphaeroides* and *Blc. viridis*, and thus the high probability of introducing unwanted mutations during replacement via homologous recombination, the native *bchX*, *bchY* and *bchZ* genes were deleted in both WT and Δ*bciA* backgrounds prior to the integration of the *Blc. viridis* orthologs. The resulting strains, Δ*bchXYZ* and Δ*bciA*/Δ*bchXYZ*, were unable to synthesize BChl and therefore could not grow under photosynthetic conditions. The precursor pigments accumulated by these strains were analyzed by HPLC (Fig. 3). Both the WT and Δ*bciA* strains were able to synthesize BChl *a*, and accordingly, no precursor pigments were detected (Fig. 3, traces A,B). The Δ*bchXYZ* strain accumulated a species of Chlide, identifiable by the presence of a Q_y_ absorption band at ~ 660 nm; however, this species had a blue-shifted Soret band (Fig. 3, trace C). The Chlide species accumulated by Δ*bciA*/Δ*bchXYZ* also had a blue-shifted Soret band (Fig. 3, trace D), although this shift was less pronounced than that of the pigment from Δ*bchXYZ*. The step in BChl biosynthesis subsequent to C7 = C8 reduction is the hydration of the 3V group of BChlide, catalyzed by the gene product of *bchF* [30], yielding a hydroxyethyl (HE) group. However, it has been documented that disruption of the genes encoding COR can result in the accumulation of a chlorin with a HE group at C3 [31], [32], indicating that BchF displays relaxed substrate specificity. N22, a non-photosynthetic strain of *Rba. sphaeroides*, was found to accumulate 3HE Chlide, while the mutation was mapped to a COR*a*-encoding gene [33]. HPLC analysis of this strain indicated that the pigment had the same absorption spectrum and retention time as the pigment extracted from Δ*bchXYZ*, which we have assigned as 3HE,8E Chlide (Fig. 3, trace E). The red shift in the Soret band of the pigment accumulated by Δ*bciA*/Δ*bchXYZ*, when compared to that of 3HE,8E Chlide, can be accounted for by the lack of both 8VRs; an 8- to 10-nm shift in this band is consistent with 8V/8E substitutions of chlorins [34]. We have therefore assigned this pigment as 3HE,8V Chlide.

**Fig. 4:**
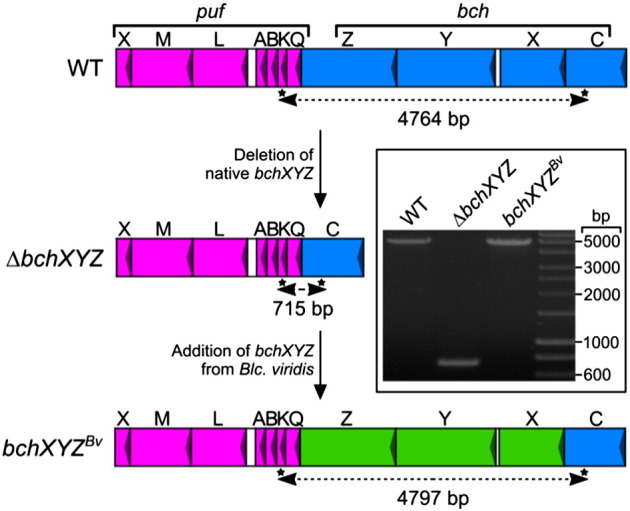
Construction of *Rba. sphaeroides* mutants designed to express the genes encoding COR*b* from *Blc. viridis.* Diagram depicting the deletion of native COR*a*-encoding genes (*bchX*, *bchY* and *bchZ*) and their subsequent replacement with orthologs from *Blc. viridis* in the region of the photosynthesis gene cluster containing RC-LH1 ORFs in the *puc* operon. Isolation of mutants was checked with colony PCR (inset) by amplifying regions between asterisks and confirmed by sequencing of the amplicons. The fragments amplified from WT, Δ*bchXYZ* and *bchXYZ^Bv^* were 4764 bp, 715 bp and 4797 bp, respectively.

**Fig. 5:**
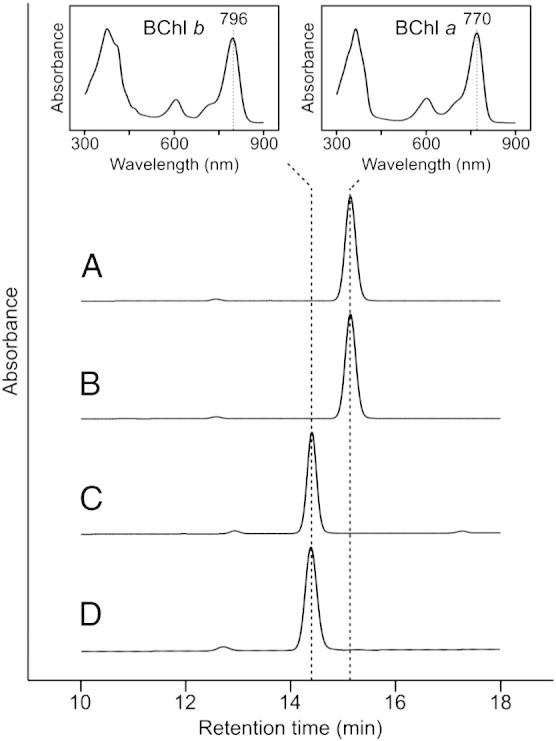
HPLC analysis of BChls extracted from *Rba. sphaeroides* strains. HPLC elution profiles of extracts from (A) WT, (B) *bchXYZ^Bv^*, (C) Δ*bciA*/*bchXYZ^Bv^* and (D) *Blc. viridis*. Retention times of 14.4 and 15.1 min, and Q_y_ absorption maxima at 796 and 770 nm (inset), are indicative of BChl *b* and BChl *a*, respectively, in the HPLC solvents. Traces are normalized to major peak height for clarity.

The COR*b*-encoding genes from *Blc. viridis* were introduced into the genomes of Δ*bchXYZ* and Δ*bciA*/Δ*bchXYZ* at the loci of the original COR*a*-encoding genes, yielding *bchXYZ^Bv^* and Δ*bciA*/*bchXYZ^Bv^*, respectively (Fig. 4). These strains, along with the WT, were grown under microoxic conditions in the dark and were tested for BChl species production by HPLC analysis (Fig. 5). WT *Rba. sphaeroides* accumulates BChl *a* exclusively (Fig. 5, trace A). We have previously demonstrated that the Δ*bciA* strain, lacking the conventional 8VR, produces BChl *a*, identical to the WT [11]. The *bchXYZ^Bv^* strain also demonstrated BChl *a* accumulation (Fig. 5, trace B). This result was unexpected since it had already been shown that COR*b* could not reduce the C7 = C8 of 8E Chlide *in vitro*, and that this form of the enzyme catalyzes ethylidene group formation [16]. However, the species extracted from the Δ*bciA*/*bchXYZ^Bv^* strain displayed a shorter retention time than BChl *a* and a red-shifted Q_y_ absorption band (Fig. 5, trace C) and was identical in these respects to BChl *b*, extracted from WT *Blc. viridis* cells (Fig. 5, trace D). These data indicate that the lack of the conventional 8VR in the COR*b*-producing strain was essential for the biosynthesis of BChl *b in vivo*. When this 8VR is present, *bchXYZ^Bv^* produces BChl *a*, albeit in low amounts, while BChl *b* is undetectable. This result suggests that the substrate for COR*b* does not bypass BciA and therefore carries an 8E group prior to C7 = C8 reduction. Although Tsukatani *et al.* [16] demonstrated that no product was formed when COR*b* was incubated with 8E Chlide *in vitro*, it is possible that the enzyme is able to reduce the C7 = C8 of 3HE,8E Chlide *in vivo*. Studies with *Rhodobacter* spp. mutants have shown that COR demonstrates substrate flexibility and can reduce Chlide species carrying a 3HE group [32], [35], [36]. It is likely that the heterologous assembly of the COR*b* complex in *Rba. sphaeroides* provides a favorable environment for enzyme activity in terms of interactions with neighboring enzymes in the BChl pathway, allowing the formation of an 8E bacteriochlorin that is subsequently converted to BChl *a*.

**Fig. 6:**
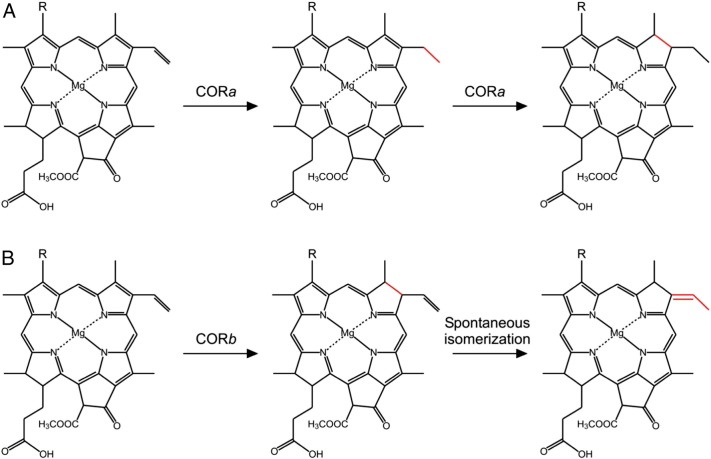
Proposed catalytic mechanisms of CORs with an 8V substrate. (A) COR*a* reduces the 8V group before reducing the C7 = C8. (B) COR*b*, having lost its 8VR activity, reduces C7 = C8 to produce an 8V bacteriochlorin which spontaneously isomerizes to the more stable *b*-type bacteriochlorin carrying an ethylidene group. R may represent either a V (HC = CH_2_) or an HE (HOC-CH_3_) group, depending on whether the pigment has undergone hydration catalyzed by the gene product of *bchF*.

The formation of the ethylidene group, found on BChl *b*, is observed in the Δ*bciA*/*bchXYZ^Bv^* strain but not in *bchXYZ^Bv^*. This result leads us to postulate that, rather than COR*b* having an ethylidene synthase function, this enzyme has lost the ability to reduce the 8V group of Chlide and that after the enzyme-catalyzed reduction of the C7 = C8, the short-lived 8V bacteriochlorin spontaneously isomerizes to the more stable *b*-type. We also hypothesize that COR*a* must catalyze the reduction of the 8V group of Chlide prior to the reduction of the C7 = C8 to prevent this isomerization and subsequent production of *b*-type bacteriochlorins in BChl *a*-utilizing organisms. These proposed mechanisms are summarized in Fig. 6.

It has been demonstrated that, when exposed to ascorbic acid photoreduction, a V group external to a newly reduced isobacteriochlorin ring is prone to “inward” double-bond migration, yielding a stable ethylidene-isobacteriochlorin [37], [38]. It has also been shown that a further inward migration is possible, with the isomerization of ethylidene groups on BChls *b* and *g* resulting in the production of chlorins with intact C7 = C8 bonds [39], [40], [41]. A similar acidic reduction may account for the presence of modified chlorins containing hydroxyl groups at C8^1^ in heliobacteria, BChl *g* producing organisms that have *b*-type CORs [42], [43]. Although it has been proposed that 8V Chlide and 3V BChlide *b* are possible substrates for this modified chlorin [43], an alternative mechanism involving hydration of the 8V group, accounting for hydroxylated Chl, and subsequent dehydration leading to ethylidene group formation has been suggested [30].

**Fig. 7:**
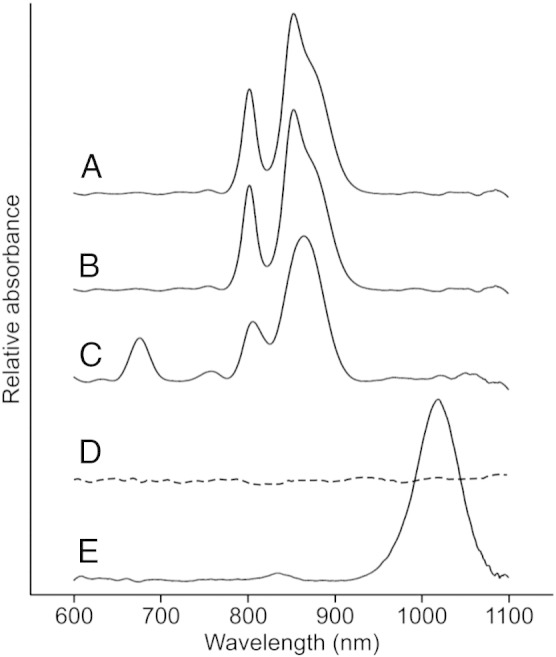
Whole-cell absorption spectra of *Rba. sphaeroides* strains. Whole cell absorption spectra of (A) WT, (B) Δ*bciA*, (C) *bchXYZ^Bv^*, (D) Δ*bciA*/*bchXYZ^Bv^* and (E) *Blc. viridis*. Spectra were recorded from cultures grown photosynthetically (solid lines) or microoxically (dashed line) when photosynthetic growth was not possible. Spectra were recorded with samples standardized by OD_710_ and absorption due to light scattering was removed by baseline subtraction from each data set.

In order to investigate the fate of BChl *b* in *Rba. sphaeroides*, strains were standardized by OD_710_ and used to inoculate fresh medium in which photosynthetic growth rates were monitored (Table 1). As expected, the WT and Δ*bciA* strains displayed similarly high growth rates, while the photosynthetic growth of *bchXYZ^Bv^* was greatly reduced. The Δ*bciA*/*bchXYZ^Bv^* strain was incapable of photosynthetic growth, demonstrating that the biosynthesis of BChl *b* in *Rba. sphaeroides* poses problems for the cellular machinery that mediates assembly of the photosynthetic apparatus. The absorption spectra of whole cells indicate that this foreign pigment is not incorporated into RC-LH1 complexes (Fig. 7, spectrum D). It is possible that the native system used to deliver pigments to the photosynthetic apparatus is incapable of handling a foreign BChl, and thus assembly of a functional unit is perturbed. Alternatively, the modification to the C8 group could lead to steric hindrance when the pigment is bound within the photosystem architecture, causing structural instability and leading to the loss of viability when grown phototrophically. The *bchXYZ^Bv^* strain exhibits limited photosynthetic growth, consistent with a reduced amount of BChl *a* synthesized. The absorption spectrum of this mutant shows that the limited amount of BChl *a* is mainly used for the assembly of RC-LH1 rather than LH2 complexes, as noticed in earlier studies where BChl biosynthesis was heavily restricted [44]. The accumulation of the 670-nm absorbing pigment, likely Chlide, indicates that the COR*b* enzyme complex in *Rba. sphaeroides* operates inefficiently, possibly due to problems interacting with other enzymes in the BChl *a* pathway.

**Table 1:** Photosynthetic growth rates of *Rba. sphaeroides* strains.

Strain	Doubling time (h)
WT	5.9 ± 0.3
Δ*bciA*	5.7 ± 0.1
*bchXYZ^Bv^*	15.3 ± 4.2
Δ*bciA*/*bchXYZ^Bv^*	–

Values are means of three biological replicates ± SD. Dash (–) indicates data could not be measured.

In WT *Rba. sphaeroides*, BChl *a* pigments within the peripheral (LH2) and core (LH1) antenna of *Rba. sphaeroides* funnel energy absorbed in the 800- to 900-nm region of the spectrum, as well as energy absorbed in the visible region absorbed by carotenoid pigments, to the RC [45], [46], [47]. In order to establish a photosystem capable of harvesting a wider range of wavelengths, it will be necessary to assemble lower-energy BChl *b-*containing RC traps that can benefit from the IR absorption of light by the BChl *b-*LH1 complexes. Thus, a mixture BChl a/*b* is required. Fluorescence-excitation experiments have shown that BChl *a*-containing LH2 from *Rhodopseudomonas acidophila* can transfer energy to the RC-LH1 complex of *Blc. viridis* in hybrid reconstituted membranes [48]. The potential ability of our strain to switch between BChl *b* and BChl *a* biosynthesis, initiated by the titration of 8VR, may permit the engineering of a system in which excitation energy is funneled from high energy BChl *a-*containing peripheral antenna (LH2) and lower energy, BChl *b*-containing core antenna (LH1) into the RC, with no overlap of the absorption bands.

## 4 Conclusions

We have engineered the biosynthesis of BChl *b* in the BChl *a*-utilizing bacterium *Rba. sphaeroides*. We hypothesize that this has been achieved by removal of the strain's ability to reduce the 8V group on a precursor of the mature pigment. The construction of this strain is an important first step in the re-engineering of photosynthesis in this bacterium.
