# Supplementary material for: Engineered biosynthesis of bacteriochlorophyll b in Rhodobacter sphaeroides
Source: Biochim Biophys Acta Bioenerg. 2014 Oct;1837(10):1611–6. doi: 10.1016/j.bbabio.2014.07.011 (PMC4331041; doi:10.1016/j.bbabio.2014.07.011)
Supplement: Supplementary file 1 — Supplementary material. [file mmc1.doc]

**Supplementary tables & figures**

| Strain/Plasmid | Properties | Source |
| --- | --- | --- |
| *E. coli* |  |  |
| JM109 | Cloning strain for pK18*mobsacB* and pBBR1MCS4 constructs | Promega |
| S17-1 | Conjugative strain for pK18*mobsacB* and pBBR1MCS4 constructs | [1] |
| *Rba. sphaeroides* |  |  |
| WT | 2.4.1 | S. Kaplan* |
| Δ*bciA* | Unmarked deletion mutant of rsp_3070 in WT | [2] |
| Δ*bchXYZ* | Unmarked deletion mutant of *bchX, bchY* and *bchZ* in WT | This study |
| Δ*bciA*/Δ*bchXYZ* | Unmarked deletion mutant of *bchX, bchY* and *bchZ* in Δ*bciA* | This study |
| *bchXYZ^Bv^*  Δ*bciA*/*bchXYZ^Bv^* | *bchX*, *bchY* and *bchZ* from *B. viridis* integrated into *ΔbchXYZ*  *bchX*, *bchY* and *bchZ* from *B. viridis* integrated into Δ*bciA*/*ΔbchXYZ* | This study  This study |
| N22 | Chemically-generated mutant in a COR*a*-encoding gene | [3] |
| *Bla. viridis* |  |  |
| WT | DSM 133 | DSMZ† |
| Plasmid |  |  |
| pK18*mobsacB* | Allelic exchange vector, *Km^R^* | J. Armitage‡ |

**Supplementary Table 1. List of strains and plasmids described in this study**

* Department of Microbiology and Molecular Genetics, The University of Texas Medical School, Houston, Texas 77030, U.S.A.

† Leibniz Institute DSMZ-German Collection of Microorganisms and Cell Cultures, Inhoffenstrasse 7B, 38124 Braunschweig, Germany

‡ Department of Biochemistry, University of Oxford, South Parks Road, Oxford OX1 3QU, U.K.

[1] Simon, R., Priefer, U. and Pühler, A. (1983) A broad host range mobilization system for *in vivo* genetic engineering: transposon mutagenesis in Gram negative bacteria. Nat. Biotechnol. **1**, 784-791

[2] Canniffe, D.P., Jackson, P.J., Hollingshead, S., Dickman, M.J. and Hunter, C.N. (2013) Identification of an 8-vinyl reductase involved in bacteriochlorophyll biosynthesis in *Rhodobacter sphaeroides* and evidence for the existence of a third distinct class of the enzyme. Biochem J. **450**, 397-405

[3] Hunter, C.N. and Coomber, S.A. (1988) Cloning and oxygen regulated expression of the bacteriochlorophyll biosynthesis genes *bch* E, B, A and C of *Rhodobacter sphaeroides*. J. Gen. Microbiol. **134**, 1471-1480

~~CCGAAACTGATCGACAGTCTGGTGATGCGGCTCGCCAAGGGCGGCGAGATCGTGCTGGCGGGCTTCTACACCGAGCCCGTCGCCTTCACCTTCGTGCCCGCCTTCATGAAGGAGGCGCGCCTGCGCATCGCTGCCGAGTGGCAGCCCGAGGACATGGTGGCCACCCGCGCGCTGATCGAGAGCGGGGCGCTTTCGCTTGCCAACCTGATCACCCACACCCGACCGGCGTCGGAGGCGGCCGAGGCCTATGCCACGGCCTTCAGCGACCCCGACTGTCTCAAGATGATCCTGGATTGGAGGGCCACCGC~~ATGACGTCCCCCGCCAGAGCGGTCTCGTTGCAGCCGATCCTTGACGCCCGTCTCAAGGCCGAAGCCGCAATCGAACCCGATCCCGTGCCCACCGGCGAGCCCGCCAAGGCGACGCAGATCATCGCGATTTACGGCAAGGGTGGCATCGGCAAGAGTTTCACGCTGTCCAACCTGAGCTACATGATGGCTCAGCAGGGCAAGAAGGTGCTGTTGATCGGCTGCGATCCCAAGGCCGACACCACCTCGCTGTTGTTCGGCGGCCGGGCCTGCCCGACCATCATCGAGACCTCGGCGAAGAAAAAGCTCGCCGGCGAAGAGGTGAAGGTCGGCGACGTCTGCTTCAAGCGCGATGGCGTGTTCGCCATGGAGCTCGGCGGCCCGGAGGTCGGGCGCGGCTGCGGCGGCCGTGGCATCATCCACGGCTTCGAGCTCCTGGAGAAGCTCGGCTTCCACGACTGGGGCTTCGACTACATTCTGCTCGACTTCCTGGGCGACGTGGTGTGCGGCGGCTTCGGCCTGCCGATCGCGCGCGATATGTGCCAGAAGGTCATCATCGTCGGCTCGAACGATTTGCAGTCGCTTTATGTCGCCAACAATGTCTGCTCGGCGGTGGAGTATTTCCGCAAGCTCGGTGGCAACGTCGGCGTCGCCGGCATCGTGATCAACAAGGATGACGGTACCGGCGAGGCGCAGGCCTTCGCGGCGGCCGCCGGCATTCCGGTGCTTTCGGCCATCCCGGCAGACGAGGATATCCGGCGCAAGAGCGCGAACTATCAGATCGTCGGCTATCCGGGCGGGGCATGGGGCTCGTTGTTCGAGGAGCTGGCGTCGGCAGTGGCGGGTGCGCCGCCGATCCGGCCGAGGCCGCTGAGCCAGGACGAGTTGCTCGCCCTGTTCAAGAGCGAGGCGGTCGGTCGCAACGTGGTGCTGGAGCCGGCGACGATCGAAGACATGTGCGGGTCGGCGGTGATCGAGAAGCCGTCGCTCGAAGTGATTTACGACACGGTGTGATCTCGATACCCTCGCGCGGCTCGATCAGGCGCTCCGTCAGATCGCCGCGCGCCAGAAGCGGAAGGGGGCAGGACGATGAGGGCTGCCAGTTACGTTCCGATCGACACCGCACGAGGCGGTGAGCCGGCGTCAGCCGGCCCGACGCCGCGCGTCGACGTTGTGGCAGGTGCGGGGGATCTGCCACGCGTCGACGTCGCTGCCATCCCCGACGGGCCCGGCTGCCGGGCCGGGTCCGTCGAGGCTTTCCGCGACTCGGTCGAAGGGCGCGGCCACGCGCCATCGTCGCCGCACGACCAGCCGCAGACGATGTGTCCGGCGTTCGGGTCGCTGCGCGTCGGTCTGCGCATGCGCCGGACCGCGACCATCCTGTGCGGCTCGGCCTGCTGCGTCTACGGTCTCACCTTCACGTCCCACTTCTATGGCGCGCGCCGCACCGTCGGCTACGTACCGTTCAGCTCCGAGACGCTGGTGACCGGCAAGCTGTTCGAGGACATCCGCGACGCGGTGCACCAGCTGGCCGACCCGGCACAATACGACGCCATCATCGTCACCAACCTGTGCGTTCCGACTGCCGCCGGCGTGCCGCTGCGCCTGTTGCCCAAGCAGGTCAACGGCGTGCGCGTGATCGGCATCGACGTGCCCGGTTTCGGCACCGGCACCCACGCCGAGGCCAAGGACATTCTCGGCGCGGCGATGCTCAACCGGGCGCGGCACGAGGCCGAGCAGGGGCCGGTGCAGGCGCCGCGCGGCGGCCGCAGCGACCGGCCGACGGTGACGCTGCTGGGCGAGATGTTCCCGGCTGACCCGGTCAACATCGGCATGCTGCTGGCGCCGCTCGGCCTGGCGGCGGGGCCGGTGGTGCCGACCCGCGAATGGCGCGAGCTGTATGCCGCGCTGGACTGCGGCGCGGTTGCGGCAATTCACCCTATTTACACCTCCGTAATTCGTGAGTTCGAAGCGGCCGGTCGCACCGTTGTGGGATCGGCCCCGGTGGGCTATGACGGCACCGCCGCGTGGCTCGACTCGATCGGCGATGCGTGCGGGGTGGCTCGGGTCACCGTCGAAGCAGCGAAAAACAAGTTGCTGCCGCCGATTCGGGCGGCGCTGGCCAACGCGCGGATCCGCGGGCGTATCACCATGTTCGGCTATGAAGGGTCGGAGTTGCTGGTGGCGCGCCTGCTGGTCGAAAGCGGGGCCGATCTTCCGTACCTCTCGACCGCCTGCCCGCGCACCCGCTGGTCGGACGCCGACCGCGAGTGGCTCGAGGCGCGGGGTGTGAAAGTTCAGTTCCGCGGAACGCACGAGCAGGACCTCGAGGCGATCGCAGAATACAAGCCAGACCTCGCCATCGGCACCACGCCGATGGTGCAGAAAGCAAAGTCGCTCGGCATTCCGTCGATTTATTTCACCAACCTGATCTCGACGCTGCCGATGTTCGGCGTCGCCGGCGCCGGCACGCTCGCCAAGCTGATGAATGCGGCGATCGGCAACAAGCCGCGGTTCGATAAGATGAGTGCGTTCTTCGCCGGTGTCGGCGAGGACGCGGAGGACGAGCGTCCCGAGTTCCGCGAAGCCTACAAGCGCCGCATCGCCGCCCAGGCTAAGCAGCGCAAGGCCGAGGAGATCGGTTGATGCTGATCCTCGACCATGATCGCGCCGGCGGCTACTGGGGGGCGGTGTACGTCTTCACGGCCGTGAAGGGGCTGCAGGTCATTATCGACGGTCCGGTCGGCTGCGAGAACCTGCCGGTGTCCGCGGTGCTGCACTACACCGACGCGGTGACGCCGCACGAGATGCCGGTGGTGGTCACCGGACTGTCGGAGGAACAACTCGGCCAGACCGGCACCGAGGAATCGATGCGGCGGGCGCACAAGACGCTCGATCCGGAGATGCCGGCGGTGGTGGTGACGGGGTCGATCGCCGAAATGATCGGCGGCGGCGTCACGCCCGAAGGCACCAATATCAAGCGGTTCCTGTCGCGCACCATCGACGAGGACCAGTGGCAGTGCTCCGACCGCGCCATGAACTGGCTGTGGACGGAGTTCGGCGCAAAGAAGGTGACGCCGCCGAAGGCGCGCAAGCCCGGCGAAAAGCTGCGGGTAAACATCATCGGCGCGGTGTATGGCACCTTCAACATTTATTCCGACCTTGCCGAAATTCGGCGGTTGGTGGAGGGAATCGGCTGCGAGGTTAACCTGGTGTTTCCGCTGGGCAGCCATCTTGCCGATGTTCCGCGGTTGTTGGATGCCGACGTCAACATCTGCATGTACCGCGAGTTCGGGCGCATGCTGTGCGAAACGTTGGAGCGGCCGTATCTGCAGGCCCCGATCGGGCTGCATTCGACGACGGCGTTTCTGCGCGCGCTCGGCGAATTGACCGGCCTCGATCCGGAGCCGTTCATCGAGCGCGAGAAGCGCACCACCATTCGTCCGCTGTGGGACCTGTGGCGGTCGATGACGCAGGATTTTTTTGGGACGTCGAGCTTCGGTATTGCTGCGAACGAGACGTACACCCGTGGACTTCGCCATTTCCTTGAGGAGGAAATGGGGCTATTGTGCAGCTTCGCTTTCTCGCGCAAGCCGGGCGTGAAGCCCGACAACGGCGCGGTTCGGCAAGCAATCCAGACCAGCATGCCGCTGATCGTGTTTGGGAGCTACAACGAACGCATGTATCTTGCGGAAGTGGGCTCGCGGGCGATTTTCATCCCCGCCTCGTTTCCGGGTGCGATCGTCCGGCGCTATACCGGAACGCCCGTTATGGGGTATGCTGGGGCAACCTATGTCGTGCAGGAAGTGTGTAATGCGCTTTACGATGCGCTGTTCAACATCCTTCCCCTCGGCACGGAACTTGATAAAGTTGAGGCGACGCCGGCCCGGCGGCATAAAGAGATGCCCTGGAACGACGACGCGAGAGCAGCTCTCGACGACTCGGTTGAGGCGCTGCCGGTTCTGATCCGCACTTCGGCCGTCAAGCGTCTGCGCGACGCGGCGGAACGGGAGGCGAGAGCAGTTGGTGAAGAGCGGGTGACGGTCGCGTGTGTTGCGCGTGCCCGTGCCGCTCTTGTAGGAGGACAGGCAGCATGA~~GCGATCATGCCGTCAACACGCCGGTCCATGCCGCCAGGGCCCACGGGCACCGAGCACCACGTGCCGAGTTCTACGTCTACTTCGCCGTCATTCTGCTGGGCGCCTTCCCGGTGGCCTTCGTGAGCTGGATCGTCTCGACGATCCGCCACCGCAGGCTTCCCAAGCGCGGCCCCTTCGCGTCCGCCTGGTTCGATGCCAAGGCGATCACGCCGCTGATTTTCCGCGCCTGACCGCAGGTCAGGTTGCGACACGCCATTCGTCGTCTCCCCAAGGGGCGGCGGATTAATCGGGAGGGCATGGTGCCTTACCGTAACCCACG~~

**Supplementary Figure 1. Annotated sequence of synthesized *Bla. viridis* *bchX*, *bchY* and *bchZ***

~~XXX~~ = homologous ends for recombination into the genome of *Rba. sphaeroides*

XXX = 3’ end of native *bchC*

XXX = *bchX^Bv^*

XXX = *bchY^Bv^*

XXX = *bchZ^Bv^*

XXX = native *pufQ*

X = base changed to remove restriction site required for subcloning

X = base changed to maintain essential upstream promoter region for *puf* operon

X = base changed to retain ribosome binding site and start codon for *pufQ*
